# Values Evolution in Transitional China: An Institutional Perspective

**DOI:** 10.3389/fpsyg.2021.792707

**Published:** 2021-11-26

**Authors:** Gong Sun, Jian Li

**Affiliations:** ^1^Business School, Changshu Institute of Technology, Suzhou, China; ^2^School of Management, Minzu University of China, Beijing, China

**Keywords:** values, evolution, China, transition, institution

## Abstract

The values are greatly affected by the social and economic environment of a country. Thus, social transformation can lead to the values evolution. China has been experiencing a huge social, political, and economic transition in the past four decades. The previous studies that explore the value changes in China mainly compare the values across the regions or generation cohorts. This research investigates the issue from an institutional perspective. Specifically, we propose that the diversification of ownership types—the essence of the economic and institutional reform since 1978 may result in value change. By surveying 327 participants from the state-owned enterprises (SOEs) and 220 respondents from the privately owned enterprises (POEs), the comparisons between SOEs and POEs on four value dimensions—individualism, power distance, risk aversion, and money orientation—were performed. The results basically support cross-vergence theory in the values evolution. The implications and limitations are presented as well.

## Introduction

Over the past 40 years, China has experienced staggering economic growth and has become a leading superpower in global economy today. Even during the coronavirus disease 2019 (COVID-19) pandemic beginning in early 2020, China has maintained GDP growth and is regarded as the engine of the world economy. It is widely agreed that the huge progress should be attributed to the opening-up policy enacted in 1978. This unprecedented social transition has intrigued scholars from various disciplines in recent decades. The researchers from social psychology and international management pay great attention to the values change in China and expect to find evidence to enrich the relevant theories.

Rokeach (1973) defined a value as “an enduring belief that a specific mode of conduct or end-state of existence is personally or socially preferable to an opposite or converse mode of conduct or end-state of existence.” The values transcend specific objects and situations as normative standards to direct the individual attitudes and behaviors (Homer and Kahle, 1988; Schwartz, 1992). It is noteworthy that management researchers often use the term “work/managerial values” (e.g., Ralston et al., 1999b,2006a; Froese and Xiao, 2012). This is because work can play a key role in one’s social life (Roe and Ester, 1999). Other scholars prefer the “cultural values” (e.g., Schwartz, 1999; Smith et al., 2002; Egri and Ralston, 2004; Kirkman et al., 2006) or human values (e.g., Rokeach, 1973; Schwartz and Bilsky, 1987; Schwartz, 1994). The essence of these concepts is identical. We use the term “values” in this study.

The convergence-divergence debate on value formation has lasted for decades (Tung, 2008). A divergence theory believes that national (traditional) culture shapes the value systems (Lincoln et al., 1978; Kelley and Worthley, 1981), whereas the convergence theory proposes that the business environment is the driving force that mainly affects the values (England and Lee, 1974; Yip, 1992). Over the controversy, Ralston et al. (1993) proposed the cross-vergence theory as an alternative and perfected the theory in the subsequent studies (Ralston et al., 1997, 1999a,b, 2006a). A cross-vergence theory maintains that neither national culture nor economic ideology (the proxy of the business environment) can independently shape the contemporary values of a society. Instead, these two factors synergistically influence the value system (Ralston et al., 1997). Specifically, the western-eastern and capitalism-socialism continuums are used to measure the national culture and economic ideology, respectively. Western culture and capitalist business environment may lead to a series of modern social values, such as individualism, self-reliance, openness, consumerism, and democracy, while the opposite is Eastern culture and socialist economic ideology. With this thread, Ralston and colleagues (Ralston et al., 1992, 1993) find that the scores of various values endorsed by the managers in Hong Kong between their counterparts in the United States and China support the cross-vergence theory as a result of the interaction of traditional Chinese culture and capitalist business environment in Hong Kong. In the 1997–2007 Decade-Awarding study of the Journal of International Business Studies (JIBS), Ralston et al. (1997) compare the values across China (Eastern socialist society), Russia (Western socialist society), Japan (Eastern capitalist society), and the United States (Western capitalist society). The results also validate the cross-vergence perspective.

In addition, the scholars examine cross-vergence theory through intra-nationally comparing the values among the regions (Ralston et al., 1996, 2018b; Koch and Koch, 2007; Zhang et al., 2007; Kwon, 2012). Specifically, the values held by people from the Chinese coastal and inland regions are always used for comparison based on the premise that the coastal areas in China are much more exposed to the Western culture than the inland areas, where the values are more traditional, although they share the same business environment. Then, an intriguing question is, what will happen if we compare the groups of people with the same national culture but distinct economic ideologies? This study explores the answer to this question from an institutional perspective.

### Institutional Perspective in Management Research

Peng et al. (2001) classified the Chinese organizational and management research into cultural school and institutional school and emphasized that the latter should be of more concern. Many researchers started analyzing the Chinese organizational issues from the institutional perspective in the 1990s (e.g., Walder, 1992, 1995; Luo, 1995; Boisot and Child, 1996; Peng and Heath, 1996; Shenkar, 1996; Guthrie, 1997).

In the past two decades, an increasing number of scholars have focused on the management issues during unprecedented social transformation in China (Tsui et al., 2004). In particular, as economic reform gave rise to the diverse types of organizations, the scholars tended to apply comparative analysis to some managerial behaviors, such as change attitudes (Lau et al., 2002; Zhou et al., 2006), promotion patterns (Zhao and Zhou, 2004), organizational citizenship behavior (Farh et al., 2004), leadership styles (Tsui et al., 2006b), and organizational cultures (Ralston et al., 2006b; Tsui et al., 2006a; Cooke, 2008). As the academic authority in this field, in their comprehensive review literature, both Peng et al. (2001) and Tsui and Lau (2002) point out that exploring how institutional and economic reform affect the cognitions and behaviors of individuals within the organizations should be a priority in future research.

According to social institutional theory, the way institutions evolve can greatly influence the social and managerial behavior in society (Hung et al., 2007). In the instructional article, Tung (2008) suggested that the researchers use the “organizational climate” construct to conduct comparative research within a country. For instance, beyond the region and generation factors, Tung et al. (2008) noted that significant variations should exist between the different sized enterprises in China. Along all these threads, we choose to empirically test how work values differ across various types of organizations, which is the epitome of Chinese institutional reform (Zhao and Zhou, 2004).

Before 1978, the state-owned enterprises (SOEs) were the predominant organizational form in China (Law et al., 2003). The government executed a “centrally planned economy” system, such as making plans for the SOEs and distributing resources and products all over the country. Under this system, the firms only needed to meet state quotas, and their profit motive was not allowed (Child and Tse, 2001). Since 1978, enriching ownership types has become the essence of economic reform. The government allowed the privately owned enterprises (POEs) to emerge in the socialist market economy (Chiu, 2005). Today, the management concept and style in the POEs are fairly professional and internationalized, quite different from those in SOEs. The present study will compare the work values between the SOEs and POEs.

### Values Investigated in This Research

The most prestigious values framework is the work of Hofstede (1980, 1991). After surveying the employees on the importance of 32 work-related statements in more than 40 branches of IBM with 116,000 employees across the world in 1967 and 1971, the author developed a framework of work values with four dimensions. These are individualism-collectivism, power distance, uncertainty avoidance, and masculinity. The first three are widely used in management and business research (Soares et al., 2007; Taras et al., 2010). Individualism-collectivism describes the relationship between the individual and the collective. The individualists emphasize personal thoughts, feelings, and needs, whereas the collectivists emphasize communal goals, norms, and obligations (Singelis, 1994). This dimension has the greatest influence on the social and business behavior (Sun et al., 2014b). For instance, it affects the work-related behaviors, such as reward allocation, conflict management, reward allocation, preferences for leadership styles, group-related attitudes, organizational commitment, organizational citizenship behavior, and many others (Kirkman et al., 2006; Taras et al., 2010).

In addition, power distance reflects the extent of acceptance of inequality in a culture. Power distance can affect the managerial behaviors, such as communication and negotiation style, absenteeism, perceived organizational justice, job satisfaction, openness to experience, and conformity (Daniels and Greguras, 2014). Uncertainty avoidance identifies the extent of tolerance for ambiguity and risk. It relates to change management, cooperative strategies with other firms, and enterprise innovation (Taras et al., 2010). However, the dimension actually incorporates the subdimensions of risk aversion and ambiguity intolerance (Sharma, 2010). In their study, Froese and colleagues (Froese and Xiao, 2012; Froese, 2013) use the concept of risk aversion, which reflects unwillingness of an individual to take risks, instead of ambiguous intolerance to analyze work values in the East Asian countries. The last dimension—masculinity—refers to how a group or a society stresses the masculine values, such as assertiveness, performance, and success. This dimension has few relations with the managerial behaviors, except for the attitudes toward group activities and cooperation (Kirkman et al., 2006; Taras et al., 2012).

The current study mainly follows the framework of Hofstede but makes some adjustments. Specifically, we use risk aversion rather than uncertainty avoidance to ensure that the dimension is well conceptualized. Moreover, we replace masculinity with money orientation, which refers to inclinations of the employees toward money (Tang and Chiu, 2003; Yang and Stening, 2013). This is because the latter more clearly represents success than masculinity and prevalent social and work value in East Asia (Froese and Xiao, 2012; Yang and Stening, 2013). Finally, the dimensions of individualism, power distance, risk aversion, and money orientation are incorporated in this study.

The SOEs perform paternalistic and patriotic management with strong socialist character (Cooke, 2008). In addition to the salaries, the organizations provide various benefits, such as an allowance for commuting, communication, housing, and healthcare. Under these circumstances, the employees feel the workplace is like their family, and turnover rate of employees is low. Socialism stresses equalitarianism and collectivism (Yang and Stening, 2013). Individualism and competition are not encouraged in the SOEs. A doctrine in SOEs is keeping low key and maintaining harmony with others. Moreover, the SOEs strengthen the socialist and collectivist ideologies through political study and promotion patterns (Zhao and Zhou, 2004; Cooke, 2008). In their organizational culture research, Xin et al. (2002) also proposed that SOEs stress the belief in selfless sacrifice to the country and society.

On the other hand, the POEs must be profit-oriented to survive the fierce competition, which is a feature of a market economy. They mainly follow the operational styles and management systems of modern enterprise institutions. They often lack the policy resources and sometimes even confront market barriers and discrimination. Without any policy protection, they are more used to competing with the other companies in an uncertain environment. For the employees in POEs, unlike the life-long “iron bowl rice” jobs in SOEs, only good performance can help them secure work. Most of them are contractual, and job-hopping for career development or salary increases are common. Thus, they emphasize competition, self-expression, and risk-taking. In addition, the POEs are always more exposed to the latest manufacturing and management techniques. Therefore, although Eastern culture still plays the dominant role in their values system, they are greatly exposed to market economy.

In summary, as the POEs function in a more capitalist business environment and have more contact with the modern management techniques, the employees in POEs might experience more cross-vergence effects than their counterparts in SOEs, which will lead to value discrepancies between them. Thus, we propose the hypotheses below.

H1: The POEs will score higher on individualism than the SOEs.

H2: The POEs will score higher on power distance than the SOEs.

H3: The POEs will score lower on risk aversion than the SOEs.

H4: The POEs will score higher on money orientation than the SOEs.

## Methods

This research collected data by surveying staff in several companies in Jiangsu Province—the most economically developed region of China. A cover letter assured them that their participation was voluntary and that their responses would be confidential. The respondents were from several industries, such as finance, manufacturing, and information technology. Finally, 547 valid samples were collected (327 from the SOEs and 220 from the POEs). Among the valid sample, there were332 men, accounting for 60.1%; 365respondents were under 40 years old, accounting for 66.7%; and majority of the respondents (95.1%) had a bachelor’s degree or above.

### Measures

This study adopts established scales to measure individualism, power distance, risk aversion, and money orientation. To keep the survey at a reasonable length and satisfy the conditions for latent construct measurement, the study used 4–6 items to measure each construct. This is supported by Diamantopoulos and Winklhofer (2001, p. 272): “The excessive number of indicators is undesirable because of both the data collection demands it imposes and the increase in the number of parameters when the construct is embedded within a broader structural model.” Five items from Singelis (1994) were used to measure individualism. A sample item is “My personal identity independent of others, is very important to me.” The Cronbach’s α is 0.789. We used six items from Dorfman and Howell (1988) to measure power distance. A sample item is “Managers should make most decisions without consulting subordinates.” The Cronbach’s α is 0.852. Four items from Gomez-Meijia and Balkin (1989) were adopted to measure risk aversion. A sample item is “I am not willing to take risks when choosing a job or a company to work for.” The Cronbach’s α is 0.827. We used four items from Tang and Chiu (2003) to measure money orientation. A sample item is “Money is important.” The Cronbach’s α is 0.911. Translation and back-translation methods were used to confirm that the Chinese respondents easily understood the questionnaire (Brislin, 1970). All the constructs were measured with seven-point Likert scales.

## Results

We first performed a confirmatory factor analysis (CFA) with AMOS for all the latent variables to develop a robust measurement model. The results of CFA indicated an acceptable model fit: chi-square = 570.4, df = 146, chi-square/df = 3.91, CFI = 0.92, GFI = 0.90, TLI = 0.90, RMSEA = 0.07. In addition, the CFA results show that all the factor loadings are greater than the critical value of 0.5 (ranging from 0.50 to 0.89), illustrating adequate individual item reliability (Bagozzi and Yi, 1988). The composite reliabilities of all the scales were greater than 0.80, which indicated sound psychometric properties (Bagozzi and Yi, 1988). Furthermore, the average variance extracted (AVE) for three out of four measures was above 0.5 except individualism, the AVE of which was 0.45. As Table 1 shows, no correlation between any two variables exceeded the square root of their AVE, demonstrating adequate discriminant validity between each construct and any other construct (Fornell and Larcker, 1981).

**TABLE 1:** Correlation matrix.

	1	2	3	4
1. Individualism	0.45^a^			
2. Power distance	0.27***	0.51		
3. Risk aversion	−0.01	0.46***	0.56	
4. Money orientation	0.20***	−0.10	0.10	0.73

***p < 0.001.

*^a^Numbers on the diagonal show the AVE.*

To test whether the values in the SOEs and POEs are different, a *t*-test was performed with SPSS 22.0. The results in Table 2 reveal that most of the values investigated in this study significantly differ between the two types of organizations except risk aversion. Specifically, the employees in POEs have significantly higher individualism (4.37 vs. 4.16), power distance (3.35 vs. 2.92), and money orientation (5.99 vs. 5.74), thus supporting H1-3. However, there is no significant difference between the two groups for risk aversion, although the employees from POEs have higher scores than their SOE counterparts (4.15 vs. 3.97). This might be because the employees in SOEs do not have much survival pressure. They are willing to take the risk to some extent. In general, the results demonstrate that economic ideology can play a role in formulating the values of employees.

**TABLE 2:** Values differences across types of enterprises.

Values	SOEs	POEs	*t*-values	Results
Individualism	4.16	4.37	−2.28**	SOEs < POEs
Power distance	2.92	3.35	−4.38***	SOEs < POEs
Risk aversion	3.97	4.15	−1.70	SOEs = POEs
Money orientation	5.74	5.99	−3.19**	SOEs < POEs

***p < 0.001; **p < 0.05.

Moreover, comparing the scores across these values, we find that the employees from both the SOEs and POEs highly endorse the dimension of money orientation and least agree with power distance. The former is consistent with the evidence of booming money worship and materialism in contemporary China (Yang and Stening, 2013; Sun et al., 2014a). Low power distance scores reveal that currently, most of the employees do not accept power inequality at the workplace.

## Conclusion and Implications

As the most abstract of social cognitions, the values can influence almost all the aspects of human life (Rokeach, 1973). Business decision-making heavily depends on the value systems of business professionals (England and Lee, 1974); hence, Tung (1994) advocated that Western business people should understand the values and mindset of their Chinese counterparts.

There are multiple implications of this study. First, on the theoretical side, it extends the Chinese management and organization research with the priority of testing how “reform” affects “cognitions.” Second, given the importance of values in the managerial decision-making process, it provides a theoretical foundation to understand and further study the Chinese managerial behaviors. Third, this study answers Tung’s (2008) call to focus on intra-national diversity and values change in international business/management research. Therefore, studying value variations within China, such a dynamic and heterogeneous country during the social transition period, could perfect the extant theory. As the most important theory in values evolution research, the cross-vergence construct was validated and developed through multiple applications in China. The current research improves it from a new perspective.

In practice, as China becomes the most attractive foreign investment destination, increasing business cooperation and negotiation emerge among the companies from China and those from other countries. The distinct values and managerial behaviors generate an increasing number of misunderstandings and conflicts. Tung and Miller (1990) maintained that understanding values and mindset of counterparts help build good working relationships; hence, our study is expected to help practitioners resolve the interferences. In addition, those who prepare to invest in China can benefit from knowing how to deal with the institutions of a country and manage the local workforces. Last, the Chinese SOEs have recently frequently participated in overseas collaborations and acquisitions. Therefore, through the article, the companies from other regions may better understand their unacquainted partners.

### Limitation and Future Research

This study has some limitations. First, we only collected data from one province in China. Considering the heterogeneity of values across China (Ralston et al., 1996, 2018b; Zhang et al., 2007; Kwon, 2012), future research should attempt to replicate the study in multiple regions and test the interactive effect of ownership types and regions on values. Second, the values incorporated in this research are limited. Future studies may include other value dimensions, such as locus of control, dogmatism, Machiavellianism, and others. Third, the data collected in this study are cross-sectional. A longitudinal study should be conducted to better investigate how values evolve across different times (e.g., Ralston et al., 2018a).

## Data Availability Statement

The raw data supporting the conclusions of this article will be made available by the authors, without undue reservation.

## Ethics Statement

Ethical review and approval was not required for the study on human participants in accordance with the local legislation and institutional requirements. Written informed consent for participation was not required for this study in accordance with the national legislation and the institutional requirements.

## Author Contributions

GS developed the theoretical framework, worked on data analysis and manuscript writing. JL worked on literature review and manuscript writing. Both authors contributed to the article and approved the submitted version.

## Conflict of Interest

The authors declare that the research was conducted in the absence of any commercial or financial relationships that could be construed as a potential conflict of interest.

## Publisher’s Note

All claims expressed in this article are solely those of the authors and do not necessarily represent those of their affiliated organizations, or those of the publisher, the editors and the reviewers. Any product that may be evaluated in this article, or claim that may be made by its manufacturer, is not guaranteed or endorsed by the publisher.
